# The Impact and Perception of England’s Web-Based Heart Age Test of Cardiovascular Disease Risk: Mixed Methods Study

**DOI:** 10.2196/39097

**Published:** 2023-02-06

**Authors:** Victoria Riley, Christopher Gidlow, Sophia Fedorowicz, Catherine Lagord, Katherine Thompson, Joshua Woolner, Rosie Taylor, Jade Clark, Andrew Lloyd-Harris

**Affiliations:** 1 Centre for Health and Development Staffordshire University Stoke-on-Trent United Kingdom; 2 Office for Health Improvement and Disparities London United Kingdom

**Keywords:** heart age, cardiovascular disease, CVD prevention, web-based risk assessment, CVD risk, qualitative research, cross-sectional design, cardiology, risk assessment, cardiovascular risk, heart health, user perception, risk knowledge, engagement, web-based

## Abstract

**Background:**

It is well documented that individuals struggle to understand cardiovascular disease (CVD) percentage risk scores, which led to the development of heart age as a means of communicating risk. Developed for clinical use, its application in raising public awareness of heart health as part of a self-directed digital test has not been considered previously.

**Objective:**

This study aimed to understand who accesses England’s heart age test (HAT) and its effect on user perception, knowledge, and understanding of CVD risk; future behavior intentions; and potential engagement with primary care services.

**Methods:**

There were 3 sources of data: routinely gathered data on all individuals accessing the HAT (February 2015 to June 2020); web-based survey, distributed between January 2021 and March 2021; and interviews with a subsample of survey respondents (February 2021 to March 2021). Data were used to describe the test user population and explore knowledge and understanding of CVD risk, confidence in interpreting and controlling CVD risk, and effect on future behavior intentions and potential engagement with primary care. Interviews were analyzed using reflexive thematic analysis.

**Results:**

Between February 2015 and June 2020, the HAT was completed approximately 5 million times, with more completions by men (2,682,544/4,898,532, 54.76%), those aged between 50 to 59 years (1,334,195/4,898,532, 27.24%), those from White ethnic background (3,972,293/4,898,532, 81.09%), and those living in the least deprived 20% of areas (707,747/4,898,532, 14.45%). The study concluded with 819 survey responses and 33 semistructured interviews. Participants stated that they understood the meaning of high estimated heart age and self-reported at least some improvement in the understanding and confidence in understanding and controlling CVD risk. Negative emotional responses were provoked among users when estimated heart age did not equate to their previous risk perceptions. The limited information needed to complete it or the production of a result when physiological risk factor information was missing (ie, blood pressure and cholesterol level) led some users to question the credibility of the test. However, most participants who were interviewed mentioned that they would recommend or had already recommended the test to others, would use it again in the future, and would be more likely to take up the offer of a National Health Service Health Check and self-reported that they had made or intended to make changes to their health behavior or felt encouraged to continue to make changes to their health behavior.

**Conclusions:**

England’s web-based HAT has engaged large number of people in their heart health. Improvements to England’s HAT, noted in this paper, may enhance user satisfaction and prevent confusion. Future studies to understand the long-term benefit of the test on behavioral outcomes are warranted.

## Introduction

### Background

Cardiovascular disease (CVD) remains the leading cause of death globally [1], with one-fourth of all deaths in England reportedly owing to heart and circulatory disease alone [2]. Communicating the risk of CVD to patients is challenging [3], and is influenced by several factors including patient understanding, health literacy, and personality traits [4]. There is evidence that patients and practitioners struggle to interpret traditional risk formats such as short-term percentage risk scores that are used to communicate risk [5-9], which limits their potential to encourage individuals to adopt CVD risk–reducing behaviors [5]. In recent years, other CVD risk formats, including heart age calculators, have been developed to support health care professionals with CVD risk communication. Heart age is a reflection of lifetime risk, whereby an individual’s chronological age is compared with someone of the same age, sex, and ethnicity but with optimum modifiable risk factors [10]. If an individual has ≥1 risk factors (ie, cholesterol level and blood pressure) that are less than optimal, their heart age will be higher than their chronological age. There is evidence that use of heart age improves risk perception and recall and is more emotionally impactful [10-22], compared with other risk communication methods, such as percentage risk scores.

A web-based version of the Joint British Societies–derived heart age test (HAT) [23], developed by Public Health England, British Heart Foundation, Joint British Societies for the prevention of cardiovascular disease, and NHS Digital, was first introduced in 2015, known as the HAT [24]. The HAT is freely accessible on the National Health Service (NHS) website [25] and can be used to identify CVD risk among people aged >30 years who do not have preexisting CVD. The test was created to raise awareness and increase understanding of CVD risk, provide information and direct individuals to resources, improve health literacy, and encourage individuals to take up the offer of an NHS Health Check (vascular risk assessment offered to those aged 40-74 years who have not been diagnosed with CVD, kidney disease, dementia, and diabetes) [26]. Early assessment of HAT use shows >500,000 completions between February 2015 and July 2015, broadly representing the population demographic of England [24]. Other heart age calculators have also been developed, which have been used by millions of individuals worldwide. These include the heart age tool developed by Unilever, accessed across 13 countries between 2009 and 2011 [27]; *Your Heart Forecast*, used to promote clinical guidance in New Zealand [28]; Framingham version of heart age, used to identify population estimates of heart age in the United States and China [29,30]; and Australia’s heart age calculator [31], created during a national consumer awareness campaign in 2019.

Despite their popularity, it has been noted that web-based CVD risk calculators (ie, including heart age calculators) produce variable risk estimates, often fail to disclose the models upon which they were based, can result in limited understanding and concern regarding CVD risk, and lead to poor behavioral intentions [13,32]. There is also the risk that heart age calculated based on incomplete data owing to poor user awareness of physiological risk information is also poor [24,27] and can lead to underestimation or overestimation of CVD risk [19,27], doubts about the credibility of the risk calculator [13,33], and unnecessary primary care visits and clinical testing [31,34]. Evaluation of Australia’s heart age calculator suggested that it provoked a positive emotional response and led to self-reported health behavior change (ie, improvement in diet, physical activity, and weight loss) and clinical checks for more than half of the survey respondents [31]. Despite such suggestions that communication of chronic risk through *age concepts* may improve behavioral outcomes over percentage risk scores (ie, low blood pressure, change in cholesterol level, and intentions to improve diet and increase exercise), a recent systematic review concluded that evidence remains limited [13,20].

There has been little research on England’s web-based HAT, despite its apparent popularity based on data published in 2016 [24]. This study provides a necessary contribution to understand its use and possible impact.

### Aim

The aim of the study was to understand who is accessing England’s HAT and its effect on knowledge and understanding of CVD risk, future intentions toward health behavior change, and potential engagement with primary care services.

## Methods

### Design

A mixed methods design was used in this study. Data were collected in one of the following three ways: (1) HAT user data (aggregate data provided by Public Health England), (2) open web-based survey, and (3) semistructured interviews.

### Ethics Approval

Ethics approval was obtained from Staffordshire University (reference SU20-085/096/101). The procedures followed were in accordance with the ethical standards of the institutional committee and with the Helsinki Declaration of 1975. Participants were informed that completion of the web-based survey was deemed informed consent. Written and audio-recorded verbal consent was obtained from those who participated in a follow-up interview.

### Settings and Participants

As it is a web-based tool, there is no geographical constraint on who can access the test; therefore, HAT user data cannot be attributed to only those living in England.

Participants were adults (aged ≥30 years) who had completed the HAT. The study setting was England, the United Kingdom. The test was completed via the web [25]. According to the purposes of the study, participants who completed the web-based survey and follow-up interviews were required to be living in England at the time of participating in the study.

### Processes and Procedures

#### Recruitment

Information about the study (including the purpose, estimated time to complete the survey, data storage, and details about the research team) and how to participate was presented to potential participants before survey completion, through a URL shared via several web-based platforms (ie, Facebook, Twitter, university website [used by university staff and prospective and preexisting students and academics], and the university’s Centre for Health and Development newsletter). A pop-up was also created by NHS Digital and displayed on the HAT results page (on the NHS website; used by the general population to obtain health information) to promote the study. The survey was voluntary. Survey respondents were invited to provide their contact information at the end of the web-based survey if they wished to participate in a follow-up interview to discuss their experience in more detail. Contact information was stored separately from the data collected in a secure laptop and destroyed after the study was completed. Both the web-based survey and interview were incentivized to encourage individuals to participate (through a prize draw and individual retail vouchers, respectively). Owing to COVID-19 restrictions and the geographical distribution of participants, interviews were conducted via telephone.

#### Data Collection and Analysis

##### User Data

Aggregate quantitative data about HAT users were obtained from Public Health England by the research team in March 2021. The data were summarized to profile users of the HAT.

##### Open Web-Based Survey

Data were collected between January 2021 and March 2021, through a web-based survey, whereby participants were asked to complete the HAT before answering questions about their experience and impact of the test, future behavior intentions, and demographic profile. Nonvalidated survey questions were created based on the aim of the study, through discussions among authors and the project steering group, and based on a previously unpublished survey created by Public Health England to understand the value of the HAT. Questions were presented across 5 pages (on average, 5 items per page), in the same order for all participants, but using survey logic to omit certain questions where appropriate. Completeness checks were not conducted, and participation and completion rates were not recorded and therefore cannot be reported.

To meet the objectives of the evaluation, responses were analyzed descriptively, and a summary of the findings is outlined.

##### Follow-up Interviews

A subsample of survey respondents participated in a semistructured, one-to-one, telephone interview to talk about their experience and the effect of the tool on future behavior intentions. An interview topic guide was used to support the discussion; it was informed through discussions among authors and the HAT steering group (including colleagues from NHS Digital, University College London, and British Heart Foundation) and a previously unpublished survey created by Public Health England to understand the value of the HAT. All participants who completed the interview were offered a web-based retail voucher worth £20 (US $25) in appreciation of their time. Interviews were audio-recorded following participant consent and transcribed verbatim.

Data were analyzed using inductive, reflexive, thematic analysis [35,36]. This was appropriate for this type of inquiry and the size of the sample [37] and allowed for purposive sampling (from the subsample of survey respondents who expressed interest in participating in an interview), such that the interview sample was broadly reflective of the typical HAT user population (based on age, sex, ethnicity, and deprivation). Processes followed those described by Braun and Clarke [35]. Overall, 2 researchers (first and third authors) familiarized themselves with the data through extensive reading before preliminary codes and themes were identified. A subsample of transcripts (1 in every 5 transcripts; 20% overall) were independently dual coded by both researchers to check the reliability of coding. Dual-coded transcripts were manually checked for discrepancies and indicated excellent coding consistency. Both researchers reviewed all preliminary codes before agreeing on initial themes. Themes were checked to ensure that they were data-driven and discussed with the second author before being finalized.

#### England’s HAT

England’s web-based HAT is based on the Joint British Societies’ risk calculator [23]. The calculator’s algorithm uses QRISK data to estimate individual 10-year CVD risk, lifetime risk, and heart age. Users are required to input information about their age, sex, ethnicity, postcode (to derive deprivation estimate), smoking status, height, weight, blood pressure, cholesterol level, family history of CVD, and other information about their current health status (eg, diagnosis of type 2 diabetes mellitus and rheumatoid arthritis). This information is used to estimate and present heart age and age at first CVD event (ie, CVD event–free survival age; Multimedia Appendix 1).

Heart age is calculated by comparing the user with someone of the same sex and ethnicity but with no individual elevated risk factors (ie, blood pressure, cholesterol level, and family history of CVD). If the user is unable to provide information about their blood pressure and cholesterol level, UK national averages are included to calculate their risk. Following the results, the test also provides the user with advice tailored to the individual’s risk factor profile (eg, smoking status, weight, cholesterol level, and blood pressure; refer to Multimedia Appendix 2 for an example screenshot of risk-tailored information and advice around smoking presented in HAT output).

## Results

### User Data

Between February 2015 and June 2020, there were 4,898,532 calculated HAT cases (the test can be completed more than once by the same individual). Users were most commonly men (2,682,544/4,898,532; 54.76%), aged between 50 and 59 years (1,334,195/4,898,532; 27.23%), and classified as having a White ethnic background (3,972,293/4,898,532; 81.09%). Ethnicity data showed that more cases were recorded as Indian or other ethnic background than any other minority ethnic group captured by the HAT. This is broadly representative of the national population aged between 30 and 90 years in England (sex [female]: 18,426,236/35,715,368, 51.59%; age [most commonly aged between 50 and 59 years]: 7,578,112/35,715,368, 21.22%) [38] and ethnicity data for England and Wales (ethnicity: 86% White, 7.5% Asian, 3.3% Black African or Black Caribbean, 2.2% mixed, and 1% other) [39]. Cases by deprivation based on the Index of Multiple Deprivation (IMD) 2019 (where quintile 1 [Q1] is the most deprived) [40] indicated more HAT completions among those living in the least deprived areas (quintile 5 [Q5]: 707,747/4,898,532; 14.45%), but there was representation across the strata (quintile 4 [Q4]: 611,793/4,898,532, 12.49%; quintile 3 [Q3]: 521,251/4,898,532, 10.64%; quintile 2 [Q2]: 402,331/4,898,532, 8.21%; and Q1: 267,897/4,898,532, 5.47%).

Calculated heart age was typically estimated to be 1 to 4 years older than the user’s chronological age (in 1,668,499/3,658,814, 45.60% of cases), followed by 5 to 9 years older than (684,793/3,658,814, 18.72%) and 1 year younger than or the same as the user’s chronological age (545,197/3,658,814, 14.90%), irrespective of year of completion.

HAT users often did not enter information about blood pressure or cholesterol level. More than half (n=4,898,532, 52.9%) of the cases were completed without blood pressure, more than three-fourths (n=4,898,532, 76.6%) were completed without cholesterol level, and approximately half (n=4,898,532, 48.6%) lacked both cholesterol level and blood pressure information.

### Open Web-Based Survey

The web-based survey yielded 819 responses. For those who provided demographic information (804/819, 98.2%), most were women (585/819, 71.4%), from a White ethnic background (755/819, 92.2%), and living in areas within the least deprived IMD quintile (Q5: 212/819, 25.9%; Table 1). Compared with the HAT user population, a high proportion of women and those from White ethnic background participated, but the distributions for age and deprivation were broadly representative of those who typically engage with the test.

Survey respondents understood that an estimated heart age that was older than their chronological age indicated that they were at an increased risk of a heart attack or stroke in the future (685/819, 83.6%). More respondents reportedly felt concerned or surprised by their estimated heart age than those who felt happy, satisfied, and reassured (Multimedia Appendix 3).

Approximately two-thirds (520/819, 63.5%) of the respondents stated that their heart age was higher than what they expected (Table 2). However, at least half of the respondents reported increase in their understanding of CVD risk (458/819, 55.9%), risk factors (414/819, 50.2%), and actions that can be taken to reduce risk (410/819, 50.1%) following completion of the HAT (Table 2). Similarly, approximately half of the respondents also reported increase in confidence related to understanding (453/819, 55.3%) and controlling (443/819, 54.1%) CVD risk (Table 2).

Most respondents reported that they intended to take some action following the completion of the test (Table 2). Intentions to set a goal to lose weight (374/819, 45.7%), followed by a goal to increase physical activity (302/819, 36.9%) and eat more healthily (283/819, 34.6%) were most commonly selected by respondents. The most common reason for not intending to take action was that their heart was healthy for their age and “other” (eg, COVID-19 restrictions and continuing healthy behavior adopted before completion of the test).

Acceptability of attending a preventative health assessment (ie, NHS Health Check) was high following completion of the test (624/819, 76.2%). Most respondents stated that they would probably (264/819, 32.2%) or definitely (375/819, 45.8%) engage with the test again in the future to assess their heart health. Those (144/819, 17.6%) who reported that they would probably or definitely not engage with the test again in the future reported that their estimated heart age was a lot (59/144, 40.9%) or a little higher (44/144, 30.5%) than what they expected.

**Table 1:** Characteristics of individuals who completed the web-based survey (N=819) compared with those of typical users of HAT^a^.

Characteristics			Individuals who completed the web-based survey, n (%)
**Age range (years)**			
	30-35	65 (7.9)	
	36-40	69 (8.4)	
	41-45	52 (6.3)	
	46-50	90 (10.9)	
	51-55	108 (13.2)	
	56-60	128 (15.6)	
	61-65	121 (14.8)	
	66-70	87 (10.6)	
	71-74	47 (5.7)	
	≥75	37 (4.5)	
	Missing	15 (1.8)	
**Sex**			
	Male	219 (26.8)	
	Female	585 (71.4)	
	Missing	15 (1.8)	
**Ethnic group**			
	White	755 (92.2)	
	Indian	13 (1.6)	
	Pakistani	4 (0.5)	
	Bangladeshi	1 (0.1)	
	Other Asian	3 (0.4)	
	Black Caribbean	4 (0.5)	
	Black African	5 (0.6)	
	Chinese	3 (0.4)	
	Other	13 (1.6)	
	Prefer not to answer	3 (0.4)	
	Missing	15 (1.8)	
**Deprivation (IMD^b^ quintiles)**			
	1	75 (9.2)	
	2	115 (14.4)	
	3	148 (18.1)	
	4	170 (20.8)	
	5	212 (25.9)	
	Missing	99 (12.1)	
**Last contact with GP^c^**			
	In the past week	88 (10.7)	
	In the past month	118 (14.4)	
	In the past 3 months	150 (18.3)	
	In the past 6 months	89 (10.9)	
	In the past 12 months	94 (11.5)	
	>12 months ago	265 (32.4)	
	Missing	15 (1.8)	
**Have a longstanding illness, disability, or disorder**			
	Yes	259 (31.6)	
	No	527 (64.3)	
	Prefer not to answer	18 (2.2)	
	Missing	15 (1.8)	

^a^HAT: heart age test.

^b^IMD: Index of Multiple Deprivation.

^c^GP: general practitioner (or physician).

**Table 2:** Expectations, understanding, confidence, and actions following completion of the heart age test (N=819).

Categories, statements, and responses				Values, n (%)
**Expectations**				
	**Estimated heart age**			
		A lot higher than expected	238 (29.1)	
		A little higher than expected	282 (34.4)	
		As expected	162 (19.8)	
		A little lower than expected	59 (7.2)	
		A lot lower than expected	16 (1.9)	
		No expectation	62 (7.6)	
**Understanding (following completion of the heart age test, has it helped to understand more about...)**				
	**Your chance of having a heart attack or stroke**			
		Not at all	99 (12.1)	
		About the same as before	262 (31.9)	
		A little more	240 (29.3)	
		Somewhat more	130 (15.9)	
		A lot more	88 (10.7)	
	**Factors that can increase your chance of having a heart attack or stroke**			
		Not at all	67 (8.2)	
		About the same as before	338 (41.3)	
		A little more	202 (24.7)	
		Somewhat more	127 (15.5)	
		A lot more	85 (10.4)	
	**Factors that can reduce your chance of having a heart attack or stroke**			
		Not at all	75 (9.2)	
		About the same as before	333 (40.7)	
		A little more	201 (24.5)	
		Somewhat more	127 (15.5)	
		A lot more	83 (10.1)	
	**Actions you could take to reduce your chance of having a heart attack or stroke**			
		Not at all	92 (11.2)	
		About the same as before	317 (38.7)	
		A little more	179 (21.9)	
		Somewhat more	140 (17.1)	
		A lot more	91 (11.1)	
**Confidence (following completion of the heart age test, how confident are you...)**				
	**Understand what risk factors could increase your chance of having a heart attack or stroke**			
		Not at all	32 (3.9)	
		About the same as before	334 (40.8)	
		A little more	93 (11.4)	
		Somewhat more	130 (15.9)	
		A lot more	230 (28.1)	
	**Understand how to change your chance of having a heart attack or stroke**			
		Not at all	48 (5.9)	
		About the same as before	315 (38.5)	
		A little more	114 (13.9)	
		Somewhat more	150 (18.3)	
		A lot more	192 (23.4)	
	**Have control over your chance of having a heart attack or stroke**			
		Not at all	59 (7.2)	
		About the same as before	317 (38.7)	
		A little more	123 (15)	
		Somewhat more	168 (20.5)	
		A lot more	152 (18.6)	
	**Can reduce your chance of having a heart attack or stroke**			
		Not at all	48 (5.9)	
		About the same as before	311 (37.9)	
		A little more	132 (16.1)	
		Somewhat more	165 (20.1)	
		A lot more	163 (19.9)	
	**Have the skills or support you need to reduce your chance of having a heart attack or stroke**			
		Not at all	75 (9.2)	
		About the same as before	327 (39.9)	
		A little more	119 (14.5)	
		Somewhat more	164 (20)	
		A lot more	134 (16.4)	
**Actions**				
	**Having found out your estimated heart age, do you intend to take any of the following actions...**			
		Blood pressure check by a GP^a^, nurse, or pharmacist	127 (15.5)	
		Check my blood pressure myself (home blood pressure monitor)	211 (25.8)	
		Book an appointment to get my cholesterol levels checked	236 (28.8)	
		Set a goal to attempt to quit smoking	17 (2.1)	
		Set a goal to lose weight	374 (45.7)	
		Set a goal to eat more healthily	283 (34.6)	
		Set a goal to get more active (i.e., going for a walk a day)	302 (36.9)	
		Look for more information about heart health	111 (13.6)	
		I do not intend to take any action	146 (17.8)	
		Something else	107 (13.1)	

^a^GP: general practitioner (or physician).

### Follow-up Interviews

#### Overview

Semistructured telephone interviews were conducted with a subsample of survey respondents (33/819, 4%; mean duration 21, SD 6 minutes). Most participants were aged between 51 and 60 years (10/33, 34%), women (19/33, 58%), from a White ethnic background (27/33, 82%), and living in areas ranked among the most deprived 50%, nationally (19/33, 58%; Table 3). The average duration between completion of the test and the interview was 8 (SD 3; range 2-13) days.

Analysis of interview data produced 4 themes: *emotional response to estimated heart age*, *perceived understanding of CVD risk*, *perception of the HAT*, and *making a change?* Each theme is examined in turn and evidenced by interview transcripts (eg, each extract is labeled to indicate participant number, age, sex, IMD quintile, and ethnicity).

**Table 3:** Interview participants’ characteristics (n=33).

Characteristics			Values, n (%)
**Sex**			
	Male	14 (42)	
	Female	19 (58)	
**Age range (years)**			
	30-35	1 (3)	
	36-40	5 (15)	
	41-45	4 (12)	
	46-50	2 (6)	
	51-55	5 (15)	
	56-60	6 (18)	
	61-65	4 (12)	
	66-70	3 (9)	
	71-75	2 (6)	
	>75	1 (3)	
**Ethnic group**			
	White	27 (82)	
	Ethnic minority^a^	6 (18)	
**Deprivation**			
	Most deprived (IMD^b^ 1-5)^c^	14 (42)	
	Least deprived (IMD 6-10)^c^	19 (58)	
**Last contact with GP^d^**			
	In the past week	4 (12)	
	In the past month	5 (15)	
	In the past 3 months	8 (24)	
	In the past 6 months	3 (9)	
	In the past 12 months	4 (12)	
	>12 months ago	9 (27)	
**Have a longstanding illness, disability, or disorder**			
	Yes	15 (45)	
	No	18 (55)	

^a^Includes those from Chinese, Indian, Black Caribbean, and other ethnic background.

^b^IMD: Index of Multiple Deprivation.

^c^IMD 1=most deprived to IMD 10=least deprived.

^d^GP: general practitioner (or physician).

#### Emotional Response to Estimated Heart Age

Following completion of the HAT, many participants were “a little bit surprised” when their result did not equate to expectations “because [they were] really active, [they] do a lot of exercise” (participant 25; aged 36-40 years; female; Q5; White) and “because [their] blood pressure is good, [their] weight is good” (participant 24; aged 41-45 years; female; Q5; White). Some of these participants felt frustrated that they “didn’t see [their] biological age” (participant 32; aged 51-55 years, male; Q2; ethnic minority) as it did not “fit with [their] experience of most people [their] age” (participant 7; aged 56-60 years; female; Q4; White). Others considered the estimated heart age to be a “real wake-up call” (participant 21; aged 66-70 years; female; Q2; White) and “a bit of a boost to say actually ‘yeah I do need to understand these levels’...I could do better with my own lifestyle” (participant 9; aged 30-35 years; male; Q3; White).

Some participants were “pleasantly surprised that [they weren’t] more unhealthy” (participant 4; aged 36-40 years; female; Q5; White). This was owing to recognition of lack of engagement in healthy behaviors:

I don’t do much exercise as I used to, or I would like to.

Participant 12; aged 41-45 years; male; Q3; White

Those who received an estimated heart age equal to or lower than their chronological age found that their result “was actually quite pleasing” (participant 10; aged 61-65 years; male; Q3; White) and it “reassured [them that] ‘oh there is a point to [a healthy lifestyle]’” (participant 11; aged 71-75 years; male; Q5; White) choosing to “los[e] some weight” (participant 5; aged 46-50 years; male; Q5; White) before taking the test.

In summary, participants reported both positive and negative emotional responses following completion of the HAT, particularly when their result did not meet expectations. For some, the test served as a wake-up call and encouraged them to re-evaluate their behavior.

#### Perceived Understanding of CVD Risk

Participants perceived to have a good understanding of their estimated heart age, with some suggesting that the test indicated their heart was older than their chronological age:

...Basically, I am a 79-year-old person.

Participant 20; aged 66-70 years; male; Q2; White

Those with an estimated heart age older than their chronological age understood that “there is obviously a little bit more [they] could do to look after [themselves]*”* (participant 25; aged 36-40 years; female; Q5; White), whereas those with an estimated heart age equal to their chronological age thought that their “behaviour, what [they are] eating, doing, isn’t making [their] heart necessarily any worse” (participant 4; aged 36-40 years; female; Q5; White).

Understanding of CVD risk was also perceived to be high, as participants stated that they were already aware of factors that can increase their risk of a heart attack or stroke as a result of information from “social media and previous knowledge” (participant 15; aged 66-70 years; female; Q4; White) or from “family members that have had issues with their hearts*”* (participant 9; aged 30-35 years; male; Q3; White).

Results from the HAT also provide users with their CVD event–free survival age (Multimedia Appendix 1; presented in HAT as “on average, someone like you can expect to live to the age of XX without having a heart attack or stroke”). A small number of participants struggled to interpret this information:

I was predicted to die at 77.

Participant 5; aged 46-50 years; male; Q5; White

From age 53, I should be expecting to have a heart attack. That is how I read it.

Participant 32; aged 51-55 years; male; Q2; ethnic minority

Others found it difficult to determine which factors were increasing their estimated heart age:

I don’t know whether it’s the cholesterol figure I put in, that is the only thing I can think of at the minute.

Participant 8; aged 56-60 years; female; Q4; White

This was concerning for a small number of participants, and therefore, the interviewer had to explain the result to provide some reassurance.

In summary, most interview participants perceived that they had a good understanding of estimated heart age and CVD risk before completing HAT owing to information obtained from social media and personal experience. Few participants struggled to interpret CVD event–free survival age presented in HAT, which led to some concern and confusion about their results and CVD risk.

#### Perception of the HAT

Most participants thought that the HAT was “easy to use and interesting” (participant 2; aged 51-55 years; female; Q2; White) or “very clear and concise” (participant 9; aged 30-35 years; male; Q3; White). The HAT was perceived to be “quite informative*”* (participant 14; aged 51-55 years; female; Q1; White) and would be helpful to those who need to improve their health behavior, “like my mum*”* (participant 6; aged 51-55 years; female; Q5; White).

However, most comments referred to the fact that heart age was estimated from limited information:

I don’t think it had much to go on.

Participant 5; aged 46-50 years; male; Q5; White

Participants expected to be asked about other factors such as alcohol or physical activity:

[It] didn’t ask me like alcohol intake...that sort of surprise[d] me.

Participant 10; aged 61-65 years; male; Q3; White

I don’t remember there being an exercise question.

Participant 8; aged 56-60 years; female; Q4; White

Others questioned the accuracy of the test when an individual is unable to report their blood pressure and cholesterol information:

With those answers [blood pressure and cholesterol] it may have been more precise, or maybe a bit more accurate.

Participant 23; aged 61-65 years; female; Q2; ethnic minority

They are fairly important measurements to put in aren’t they?

Participant 4; aged 36-40 years; female; Q5; White

Owing to this reason, a small proportion of participants whose estimated heart age was older than their chronological age chose to “discount the whole thing because you just don’t believe it, it’s how it is, isn’t it. They have got it wrong*”* (participant 11; aged 71-75 years; male; Q5; White).

Nevertheless, some participants had already recommended the test when interviewed:

My son is 33 and I said to him you need to be doing this now. My niece, I rang her and told her and my sister.

Participant 21; aged 66-70 years; female; Q2; White

Others reported that they would recommend the test “to some, not to all, it probably depends on where I think they are at, at the time” (participant 30; aged 41-45 years; female; Q4; ethnic minority). This was mostly owing to feeling that it is not their “job [to discuss health behaviour choices with someone]...It is quite a delicate subject” (participant 16; aged 51-55 years; male; Q4; White).

In summary, participants liked the simplicity of the test, but some questioned its accuracy owing to the amount of information required and when they were unable to provide information about their blood pressure and cholesterol level. However, most participants would recommend or had already recommended the HAT to others. This implies a perceived benefit regardless of their reservations about HAT’s accuracy.

#### Making a Change?

Following completion of the HAT, most participants reported that the test prompted them to consider “doing more exercises” (participant 6; aged 51-55 years; female; Q5; White), “calorie intake” (participant 25; aged 36-40 years; female; Q5; White), and weight loss of “certainly 3[kgs]” (participant 10; aged 61-65 years; male; Q3; White). Participants also suggested that the HAT could be a catalyst to engage with primary care, for example, either “just have a check-up” (participant 9; aged 30-35 years; male; Q4; White) or “to find out [what their cholesterol level and blood pressure numbers were]” (participant 30; aged 41-45 years; female; Q4; ethnic minority). However, some questioned “whether [their] motivation [would] persist” (participant 1; aged 36-40 years; male; Q4; White) once the burden of the COVID-19 pandemic became more manageable for general practice and they could subsequently book a checkup.

Some participants reported that they had already made changes to their health behavior including “doing more regular exercise” (participant 3; aged 41-45 years; female; Q1; White) and researching “about food portions...checking calories, how much do you need*”* (participant 27; aged 36-40 years; female; Q5; ethnic minority). Only 3% (1/33) of the participants would prefer to ask a health professional if they could have their cholesterol level and blood pressure checked through a routine blood test for a preexisting condition and were surprised to learn that their blood pressure was high:

The doctor rang me...he said because you have got a rheumatoid flame up at the moment, that would put your blood pressure up...if I hadn’t done that survey I wouldn’t have had a clue.

Participant 21; aged 66-70 years; female; Q2; White

This low level of follow-up among users may be explained by the COVID-19 pandemic, as participants felt that it was “probably not the right time” (participant 30; aged 41-45 years; female; Q4; ethnic minority) to ask their GP for follow-up tests. Another participant stated that the HAT had encouraged them to reconsider their smoking habit:

It brought it home a bit more to me...it was just the test that really said to me...“Hang on [name], do you have to have a cigarette now” and that has been “no,” so it is just breaking habits.

Participant 20; aged 66-70 years; male; Q2; White

Changes to behavior were largely reported to be a result of completing the HAT and “because of [their] family history” (participant 24; aged 41-45 years; female; Q5; White) or influences from family members:

My little lad...to hear him say “you know mum that has put so many years on your [heart] which means you are going to lose those years.” That was...a big factor hearing my little boy say that.

Participant 3; aged 41-45 years; female; Q1; White

Those without intentions to change their behavior explained that it was because they did not “feel there are massive life changes to be made as a result of what was in the test*”* (participant 1; aged 36-40 years; male; Q4; White) or because “[their] heart age [was] only slightly above [their] real age” (participant 22; aged 56-60 years; male; Q3; ethnic minority). However, participants stated that they would attend an NHS Health Check following the completion of the HAT as “it would be nice to understand more about...the actual health of [their] heart” (participant 26; aged 36-40 years; female; Q5; White).

In summary, most participants had intentions to change or had already made changes to their health behavior following completion of the HAT. Those who had already taken action to improve their health before completing the test reported that it had encouraged them to maintain those changes. Although behavioral intentions and changes were reportedly owing to the HAT, most participants had already made changes to their health behavior before completing HAT, which indicates that participants were already invested in improving their health.

## Discussion

### Principal Findings and Comparison With Previous Studies

To the best of our knowledge, there is limited evidence of the effect of England’s HAT from a sample of users. With approximately 5 million completions up to June 2020, the findings suggest that there is considerable public interest in heart health. Overall, users who engaged with the test were most commonly men, aged between 50 and 59 years, classified as having White ethnic background, and living in the least deprived areas, similar to a previous descriptive study published in 2016 [24]. This contrasts with other heart age calculators that have typically reported high proportions of female users [27,31]. This may be explained by a campaign in 2018, which led to a surge in HAT engagement, particularly from men.

Analysis of the web-based survey and interview data suggested that the HAT provoked a negative emotional response when the score did not meet expectations, reflective of findings reported elsewhere [31]. Participants also stated that they understood the significance of estimated heart age being higher than their chronological age and self-reported at least some improvement in understanding of their CVD risk and confidence in understanding and controlling their CVD risk. Compared with percentage risk scores, there is evidence that heart age is more emotionally impactful and improves risk perception and recall [10-12,15-22]. However, CVD event–free survival age (presented in HAT—refer to Multimedia Appendix 1) was reportedly difficult to interpret, which led to some concern and confusion about why their estimated heart age was higher than their chronological age for some participants. There is little evidence of the impact of CVD event–free survival age, but poor understanding from both patients and practitioners has been reported elsewhere [5,41], suggesting the need for great caution and clarity when presenting risk information in this format.

Participants questioned the accuracy of the HAT, largely owing to the small amount of information required from which heart age was estimated and the implications of not knowing their blood pressure or cholesterol level to inform this estimate. Concerns that heart age can overestimate CVD risk are well reported [19,27,31,33,34] and have led to calls for caution in its application [17,34]. Nevertheless, most participants stated that they would recommend or already had recommended the HAT to others, would engage with the test again in the future, and would be more likely to take up the offer of an NHS Health Check and self-reported that they had made or intended to make changes to their health behavior (ie, lose weight, be more active, and eat more healthily) or were encouraged and motivated by the test to maintain the changes made to their health behavior.

Researchers have suggested that estimated heart age can increase motivation for individuals to make changes to their health behavior [10-12,19-22,31] and perform clinical checks [20,31]. As with Australia’s heart age calculator, participants most commonly self-reported changes or intentions to improve their diet, lose weight, and be more active following completion of the HAT [31]. However, a recent systemic review that explored the effects of heart age interventions concluded that there is limited evidence to suggest that heart age alone can lead to positive behavioral outcomes [13]. In this study, participants reported some engagement in healthy behaviors before completing the HAT, and their motivation to reduce their CVD risk also resulted from other factors including supportive family and friends and family history of CVD. Therefore, heart age calculators may be a method that can be used in combination with other behavioral strategies to encourage individuals to re-evaluate their current health behavior and to increase intentions to improve their heart health. The long-term outcomes from HAT are yet to be explored.

### Strengths and Limitations

To the best of our knowledge, this is the first study of England’s HAT to explore user experiences and intentions to action. Strengths of this study include the use of multiple data sources, which allowed for cross-validation of findings and participant experiences. The survey sample differed in some aspects but still represented the sociodemographic range of the population of England, with participants from various age, sex, ethnic background, and deprivation levels. Interview participants were purposively sampled to be representative of the typical profile of HAT users, with overrepresentation of those from ethnic minority backgrounds to ensure that a range of views and experiences were captured. A subsample of interviews was independently coded by 2 qualitative researchers, which led to a robust examination of the data.

Several limitations are acknowledged. First, deprivation could not be determined for approximately half (2,387,513/4,898,532, 48.74%) of HAT users. Users may have completed the HAT with fictitious data, and those with no postcode could reside outside of England, which could undermine assertions about the HAT user population. Second, the self-selecting sample introduces a degree of bias to be reflective of those who typically engage with digital self-checking tests (ie, ecologically valid). This may be arguably great in the interview sample, representing those who are more knowledgeable and positive about their health. However, both positive and negative views and experiences were described by participants, which suggests this had a limited influence on the findings. Third, to be representative of typical HAT users, few older participants were recruited for follow-up interviews. Owing to their age, these individuals are predisposed to an increased CVD risk, which may be likely to affect their perception of the test and future behavioral intentions. Their limited inclusion in the study may have influenced the findings. Fourth, there is underrepresentation of those living in the most deprived areas. Although this is representative of those who typically engage with the HAT, it limits the conclusions that can be drawn in this sample of individuals. Further studies are needed to understand the impact of heart age on those who are most deprived. Fifth, many participants self-reported that their intentions to change or actual changes to their health behavior resulted from completing the HAT (Table 2). However, as most participants reported at least some engagement in healthy behaviors before completing the test, these outcomes cannot be attributed to the HAT alone. Therefore, participants’ future engagement in healthy behaviors cannot be attributed to competing the HAT alone. Future studies could explore the impact of HAT on those who are not currently engaged in risk-reducing behaviors. Sixth, data were collected during a period of national lockdown (England; January 2021 to March 2021), which may have affected participant responses (ie, self-reported intentions to change or actual behavior change and access to health care services). This may have led to individuals underreporting or overreporting their intentions to change behavior and may have reduced access to health care services.

### Future Directions

Completion of England’s HAT elicited a negative emotional response when the result did not match previous risk perception. Although this served as a wake-up call for most participants, the credibility of the test was questioned by all participants who were interviewed and subsequently dismissed by some. Therefore, adequate direction to resources and more information about how estimated heart age is calculated is needed to support users who may feel confused or concerned about their result. Clear information about the accuracy of the result is also warranted, especially if the user was unable to provide physiological risk factor information (ie, blood pressure and cholesterol level).

Most participants reportedly had a good understanding of the meaning of high estimated heart age, suggesting that heart age calculators may be a good way to improve the population’s understanding of CVD risk. However, given the misinterpretation of CVD event–free survival age, great caution and clarity are needed when presenting risk information in this format. Participants also self-reported changes to their health behavior and intentions to make healthy behavior choices and engage with primary care services (ie, arrange a blood pressure or cholesterol check) upon completion of the test. However, it could not be determined if these participants were estimated to have a heart age that was older than their chronological age, as few participants shared their result during interview. Nevertheless, web-based tests such as HAT may be a good way to encourage individuals to manage their own health by self-checking their heart health. Where clinically appropriate, some users reported intending to see a health care professional for blood pressure and lipid assessments. This could support a range of incentives recently introduced in England to enable individuals aged >40 years to get their blood pressure checked.

The number of HAT completions reported here (approximately 5 million from February 2015 to June 2020) suggests considerable public interest in heart health. However, there was a pattern of underrepresentation of those living in the most deprived areas in this study, which suggests a need to further explore the extent of inequalities, regarding both reach or access and how the potential benefits are distributed across the socioeconomic strata.

### Conclusions

With approximately 5 million completions up to June 2020, findings from our evaluation of England’s HAT in a subgroup of users suggest that there is considerable public interest in heart health. The test was shown to elicit a more negative emotional response when estimated heart age did not equate to previous risk perceptions. The test reportedly led to an increased understanding of high estimated heart age and at least some improvement in understanding of CVD risk and confidence in understanding and controlling CVD risk. Despite concerns resulting from the limited information needed to complete the test or missing physiological risk factor information (ie, blood pressure and cholesterol level), participants stated that they would recommend or had already recommended the test to others, would use it again in the future to check their heart health, and would be more likely to take up the offer of an NHS Health Check and self-reported that they had made or intended to make changes to their health behavior or felt encouraged and motivated by the HAT to continue the changes made to their health behavior. However, many participants self-reported at least some engagement in healthy behaviors before completing the test; therefore, some of these outcomes cannot be attributed to the HAT alone. A web-based self-checking test such as England’s HAT may be a good way to raise awareness about CVD risk and encourage individuals to self-check their heart health and consider healthy behavior choices in combination with other behavioral strategies. However, more adequate direction to support and information about how estimated heart age is calculated and presentation of CVD event–free survival age should be considered to avoid user confusion and improve satisfaction.

## Appendix

Multimedia Appendix 1 Screenshot of heart age and cardiovascular disease (CVD) event-free survival age presented in heart age test (HAT) output.

Multimedia Appendix 2 Screenshot of risk tailored information and advice presented in heart age test (HAT) output.

Multimedia Appendix 3 Participants’ emotional response to estimated heart age.

## Abbreviations

CVD: cardiovascular disease
HAT: heart age test
IMD: Index of Multiple Deprivation
NHS: National Health Service
Q1: quintile 1
Q2: quintile 2
Q3: quintile 3
Q4: quintile 4
Q5: quintile 5

## Data Availability

The data sets generated and analyzed during this study are not publicly available because consent was not obtained from participants during data collection.
