# Supplementary figures and images for: The Impact and Perception of England’s Web-Based Heart Age Test of Cardiovascular Disease Risk: Mixed Methods Study
Source: JMIR Cardio. 2023 Feb 6;7:e39097. doi: 10.2196/39097 (PMC9983813; doi:10.2196/39097)

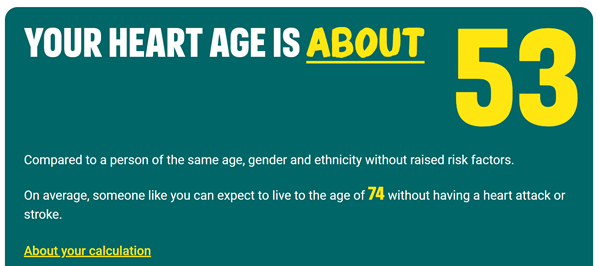

Supplement: Multimedia Appendix 1 [file cardio_v7i1e39097_app1.png]

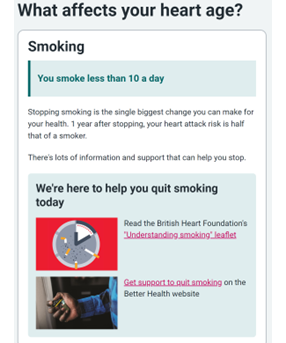

Supplement: Multimedia Appendix 2 [file cardio_v7i1e39097_app2.png]

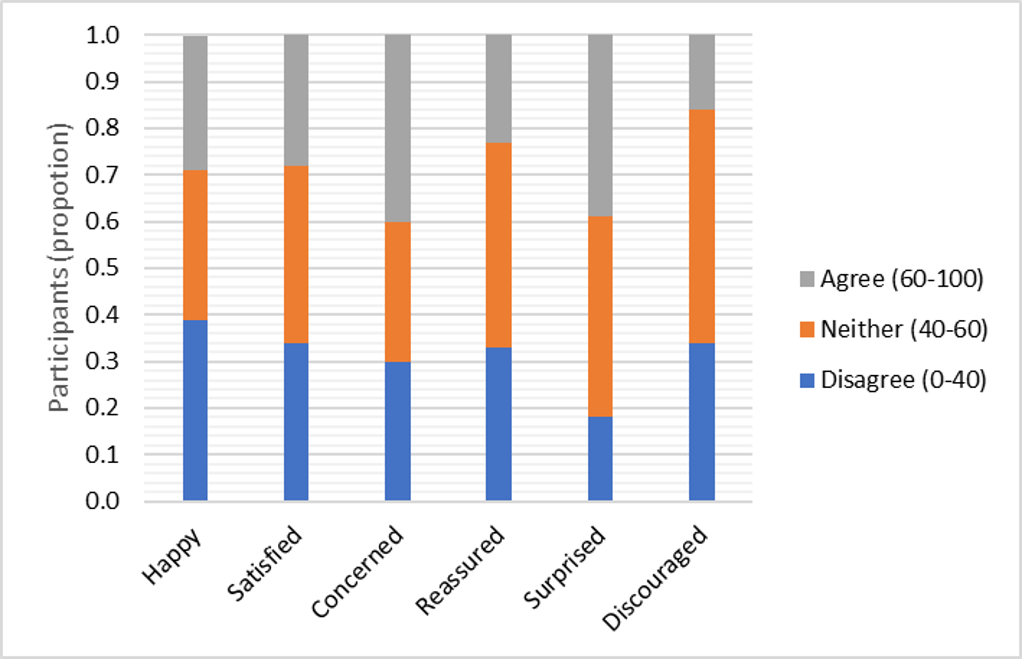

Supplement: Multimedia Appendix 3 [file cardio_v7i1e39097_app3.png]
